# Optimizing Anastomoses Technique in Orthotopic Heart Transplantation: Comparison of Biatrial, Bicaval and Modified Bicaval Technique

**DOI:** 10.3390/jcdd9110404

**Published:** 2022-11-20

**Authors:** Moritz Benjamin Immohr, Udo Boeken, Raphael Romano Bruno, Yukiharu Sugimura, Arash Mehdiani, Hug Aubin, Ralf Westenfeld, Igor Tudorache, Artur Lichtenberg, Payam Akhyari

**Affiliations:** 1 Department of Cardiac Surgery, Medical Faculty and University Hospital Düsseldorf, Heinrich-Heine-University Düsseldorf, 40225 Düsseldorf, Germany; 2 Division of Cardiology, Pulmonology and Angiology, Medical Faculty and University Hospital Düsseldorf, Heinrich-Heine-University Düsseldorf, 40225 Düsseldorf, Germany

**Keywords:** heart transplantation, warm ischemia, anastomoses, beating heart, reperfusion, cardiac surgery

## Abstract

Implantation techniques for orthotopic heart transplantation (HTx) have evolved over the centuries. Recently new approaches of modified bicaval techniques to minimize warm ischemia are gaining popularity in the literature. Between 2010 and 2022 n = 238 patients underwent HTx in our department. The recipients were retrospectively reviewed and divided regarding their anastomoses’ technique. Anastomoses were sutured either in biatrial (n = 37), bicaval (n = 191) or in a modified bicaval (n = 10) manner with suturing of the superior cava vein and A. pulmonalis anastomosis after removing the aortic cross-clamp during the reperfusion. Warm ischemia was 62 ± 11 min for biatrial, 66 ± 15 min for bicaval, but only 48 ± 10 min for modified bicaval technique (*p* < 0.001). The incidence of severe primary graft dysfunction (PGD) was comparable between biatrial (27.0%) and bicaval (28.8%) anastomoses. In contrast, in patients with modified bicaval technique PGD occurred only in a single patient (10.0%). The incidence of postoperative pacemaker implantation was 18.2% for biatrial compared to 3.0% for bicaval and 0.0% for modified bicaval technique (*p* = 0.01). The modified bicaval technique enables to decrease the crucial warm ischemia during HTx compared to both biatrial and regular bicaval techniques. Therefore, we strongly recommend bicaval anastomoses, ideally in a modified manner.

## 1. Introduction

Within the last decades, perioperative care and pharmacotherapy for patients undergoing orthotopic heart transplantation (HTx) for end-stage heart failure have tremendously evolved [1,2]. However, meanwhile surgical techniques for HTx have shown little development [3,4]. By now, the surgical technique of bicaval anastomoses is the most common one to perform for the HTx procedure, followed by a less popular biatrial technique [5,6,7,8]. Multiple studies have revealed an increased risk for tricuspid valve regurgitation as well as heart rhythm disorders requiring permanent pacemaker implantation in patients with biatrial anastomoses technique by Lower and Shumway compared to bicaval ones [5,6,7,8,9]. In contrast, bicaval technique requires suturing an additional anastomosis which prolongs the warm ischemia of the graft [5,6]. As this is a crucial parameter for graft function and postoperative survival, decreasing the warm ischemia should be one of the main goals of every transplant [10,11]. Therefore, modifications of the bicaval technique have been reported [12,13]. Hereby, anastomoses of the superior vena cava as well as the pulmonary artery and sometimes even the inferior vena cava are sutured after releasing the aortic cross-clamp in a beating-heart technique [12,13]. However, due to the worsening exposition, suturing of the remaining anastomoses after the start of the donor heart reperfusion might be more challenging especially in redo cases and for less experienced surgeons. By now, only a little evidence regarding the comparison of biatrial, bicaval and modified bicaval techniques on the outcome after HTx is reported in the literature.

In the following, we, therefore, aimed to analyze the impact of the anastomoses technique of the HTx procedure on the warm ischemia and thereby on related outcome parameters. We hypothesized that a modified bicaval technique is a feasible method to decrease warm ischemia and will therefore outclass the well-established biatrial and bicaval techniques.

## 2. Materials and Methods

### 2.1. Patients and Study Design

Between 2010 and 2022, a total of n = 238 adult patients underwent HTx in our department and were prospectively entered into an institutional database. These patients were retrospectively reviewed and assigned to three different study groups regarding the surgical anastomoses’ technique of the HTx procedure. The majority of patients (n = 191) were transplanted in the regular bicaval technique (bicaval group) which was used during the entire study period. In contrast, until 2021 in n = 37 patients biatrial Lower and Shumway technique (biatrial group) was used. The modified bicaval technique was implemented in our practice in 2021 and since then used in n = 10 cases (modified bicaval group).

### 2.2. Study Objectives and Follow-Up Period

All relevant recipient, donor and outcome parameters were examined and retrospectively compared between the study groups. While the duration of warm ischemia (period between removing the graft from the cold storage and releasing the aortic cross-clamp for reperfusion) was defined as the primary endpoint of the study, the incidence of postoperative primary graft dysfunction (PGD), perioperative morbidity, postoperative sinus rhythm and pacemaker implantations as well as 30-day and one-year survival were analyzed as secondary endpoints. Postoperative follow-up was done on regular basis every one to three months with a mean follow-up period of the whole cohort of 1077 ± 1062 days and a maximum follow-up of 4214 days. Due to the late adaptation of the modified bicaval technique, mean follow-up of this group was only 214 ± 133 days with a maximum of 368 days. Consequently, we did not examine long-term follow-up beyond the first year.

### 2.3. Surgical Procedure and Immunosuppressive Regime

**Figure 1:**
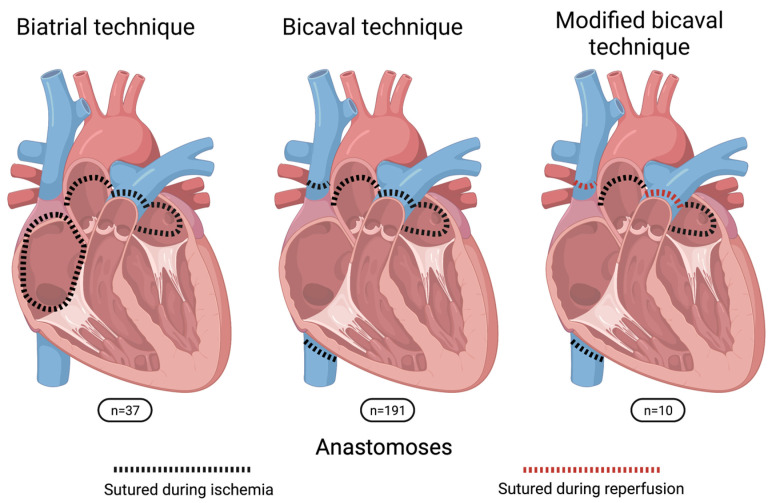
Schematic illustration of sutured anastomoses in comparison of the three different surgical techniques.

**Figure 2:**
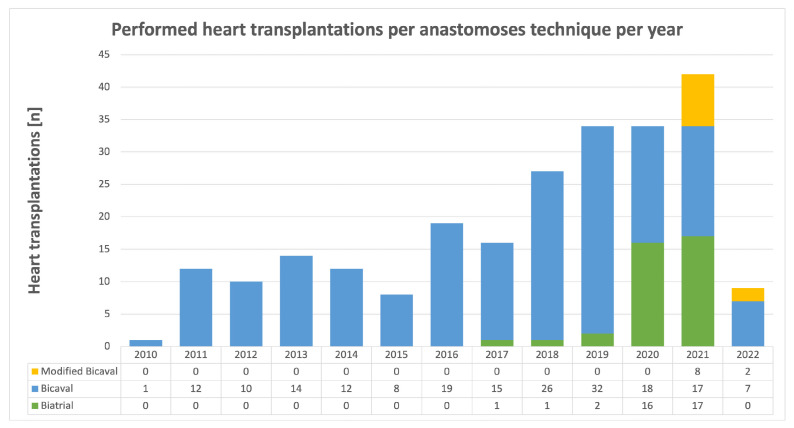
Performed heart transplantation per anastomoses technique and per year during the study period between September 2010 and March 2022. Between 2010 and 2016 only bicaval technique was performed. Modified biatrial technique was introduced in 2021.

Heart transplantation was performed in an orthotopic manner in all cases. Donor hearts were preserved in cold storage solution during transport without the usage of a specialized organ preservation system. Patients were either transplanted in biatrial, bicaval or modified bicaval technique (Figure 1). For biatrial technique, anastomoses were sutured in the following order: left atrium (LA), right atrium, pulmonary artery (PA) and aorta. For bicaval technique, the sequence was LA, inferior vena cava (IVC), superior vena cava (SVC), PA and aorta. In contrast, for the modified bicaval technique aortic cross-clamp was released after the LA, IVC and aortic anastomoses and SVC and PA were sutured during the reperfusion of the graft in beating-heart technique. The performed anastomoses technique for HTx was chosen regarding anatomical conditions of the recipients as well as the surgeons’ preferences. Figure 2 shows the performed HTx procedures per anastomoses technique and per year.

Following an institutional standard operating procedure for PGD, besides adequate inotropic and vasoconstrictive therapy, a relatively liberal regime of early implantation of veno-arterial extracorporeal membrane oxygenation (va-ECMO) and percutaneous microaxial pumps (Impella 5.0, Abiomed, Inc., Danvers, MA, USA) was conducted.

All patients followed the same immunosuppression protocol consisting of a combination therapy of tacrolimus (target level: 9–12 ng/mL), mycophenolate mofetil (target level: 1.5–4.0 µg/dL) and prednisolone. The protocol was initiated directly after the surgical procedure mostly without additional induction therapy. To examine potential graft rejection, a first endomyocardial biopsy was scheduled approximately one week after the HTx procedure. Acute graft rejection was addressed with high-dose prednisolone therapy for at least three consecutive days and in case of antibody-mediated rejection, therapy was amended by immunoabsorption or plasmapheresis, anti-T-lymphocyte IgG and intravenous IgM-enriched human immunoglobulin.

### 2.4. Statistics

Statistical analyses were calculated with SPSS Statistics 28 (IBM Corporation, Armonk, NY, USA). All results are presented as mean values with the standard deviation respectively percentages of the whole. Because of the unbalanced group sizes, normal distribution of data was not assumed. Consequently, parameters were evaluated by either non-parametric two-tailed Kruskal-Wallis tests for continuous variables, Fisher-Freeman-Halton tests for dichotomous ones or Kaplan-Meier method with log-rank test for survival analysis. In case of statistically significant results (*p* < 0.05), additional post-hoc analyses by either Bonferroni correction or pairwise Fisher’s exact test were performed.

## 3. Results

### 3.1. Preoperative Recipient Parameters

Table 1 shows the preoperative parameters of the recipients. We did not observe differences between the three study groups. Especially, parameters related to complicated surgical procedures such as body weight, body mass index, the incidence of previous thoracic surgery and present ventricular assist devices were comparable for each group. The same result was observed for concomitant diseases related to impaired post-transplant outcomes.

### 3.2. Preoperative Donor Parameters

Table 2 shows the donor data before organ recovery as listed in the Eurotransplant donor report. Relevant donor data related to impaired early graft function, such as donor age, ejection fraction, previous cardiopulmonary resuscitation, and catecholamine doses were again comparable in all groups. However, in donors of the biatrial and modified bicaval group drug abuse was more frequently observed than in the bicaval group.

### 3.3. Impact of Anastomoses Technique on Graft Ischemia and PGD

Table 3 shows the peri-procedural data with focus on the graft ischemia, PGD, blood transfusions and hospital stay. While transport time of the graft was about 150 to 160 min in all groups (*p* = 0.47), warm ischemia was significantly shorter in the modified bicaval group (48 ± 10 min) compared to both biatrial (62 ± 11 min, *p* = 0.03) and bicaval (66 ± 15 min, *p* = 0.001) techniques. In contrast, post-hoc analysis revealed no differences between the biatrial and bicaval techniques (*p* = 0.29). Cardiopulmonary bypass-assisted graft reperfusion was significantly shorter in the biatrial group (89 ± 56 min) compared to both bicaval groups (about 135 min each). Consequently, total cardiopulmonary bypass time was also shorter in the biatrial group compared to the bicaval group but not to the modified bicaval group due to the shorter cross-clamp time. Both incidences of severe PGD requiring postoperative mechanical circulatory and duration of postoperative catecholamine therapy were not significantly affected by the anastomoses technique. Nevertheless, there was a numerical trend for improved results in the modified bicaval group regarding postoperative epinephrine therapy (98 ± 44 h), norepinephrine therapy (76 ± 72 h), va-ECMO/microaxial pumps (10.0%) and successful weaning rate from va-ECMO/microaxial pump (100.0%) compared to biatrial (120 ± 92 h; 235 ± 351 h; 27.0% respectively 90.0%) and bicaval technique (153 ± 153 h; 166 ± 188 h; 28.8% respectively 70.9%). Furthermore, we observed a significantly shorter stay on the intensive and intermediate care unit for patients of the bicaval group compared to the biatrial group (post-hoc analysis *p* = 0.03). However, there was no difference between the other groups.

### 3.4. Impact of Anastomoses Technique on Postoperative Adverse Events

Table 4 shows the postoperative morbidity. We did not find a relation between the anastomoses technique and severe postoperative adverse events such as neurological events (stroke, transient ischemic attack), surgical re-exploration for thoracic bleeding, acute graft rejection and postoperative infections (sepsis, pneumonia, infective wound healing disorders).

At hospital discharge, sinus rhythm was found in about all patients of the bicaval groups, but only in about 82% of the biatrial group which was significantly impaired. As most of these patients suffered from sick sinus syndrome or atrioventricular blockage, implantation of a permanent pacemaker was required in 18.2% of biatrial patients before hospital discharge. Similar trends were also observed at the last recent follow-up visit.

### 3.5. Impact of Anastomoses Technique on Short-Term Survival

**Figure 3:**
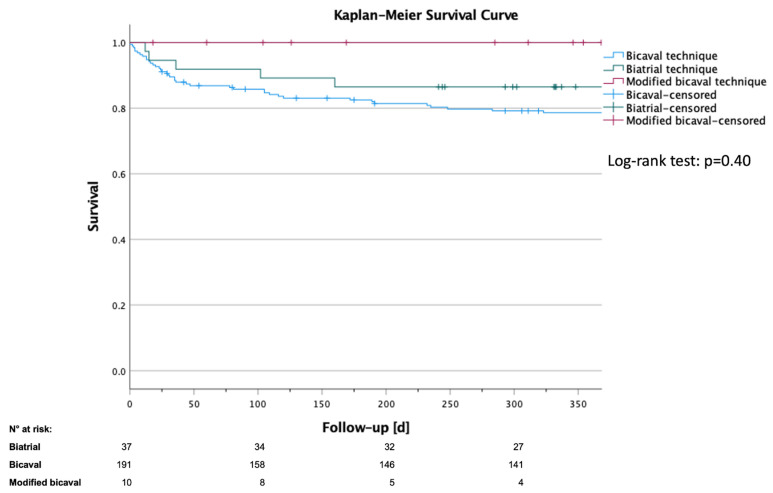
Kaplan-Meier survival curve after heart transplantation. Comparison of biatrial (n = 37), bicaval (n = 191) and modified bicaval (n = 10) anastomoses technique. Patients were censored when they reached the end follow-up period. Log-rank test: *p* = 0.40.

As indicated in the method section, survival analysis covered only the first post-operative year due to the late adaptation of the modified bicaval technique as well as the expected impact of the surgical procedure primary on short-term survival. As shown in Table 4, comparable 30-day survival of 89.4% (bicaval), 94.6% (biatrial) and 100% (modified bicaval) was observed. A similar trend was found after one year with survival ranging between 78.7% for the bicaval and 100% for the modified bicaval group. As most patients of the modified bicaval groups were censored within the first year, additional survival estimation was carried out by the Kaplan-Meier method which is shown in Figure 3. Comparison by log-rank test confirmed comparable results for all techniques (*p* = 0.40).

## 4. Discussion

In the present study, we examined the impact of the anastomoses technique on the outcome after HTx. For this purpose, we compared biatrial, bicaval and a modified bicaval technique in 238 consecutive cases. In line with the reported literature, biatrial technique was inferior to bicaval technique in regard to postoperative heart rhythm disorders requiring pacemaker implantation. Modification of the bicaval technique by suturing the pulmonary artery and superior cava vein anastomoses after releasing the aortic cross-clamp significantly decreased the warm ischemia compared to both biatrial and bicaval technique. In addition, patients with the modified bicaval technique did not experience postoperative heart rhythm disorders and had a numerically decreased incidence of PGD.

Decreasing the graft ischemia was the main goal of the introduction of the modified bicaval technique in our department. In the recent time of donor organ shortage, transport times have increased, and so-called marginal grafts are more frequently accepted [14]. To ensure optimal graft quality for every patient, multiple new technologies of graft preservation have been developed within the last decade [15,16]. However, these technologies are often more complex and expensive than standard preservation in cold storage solutions [15,16]. In contrast, decreasing the warm ischemia by enhancing the surgical technique of the HTx procedure is way easier and cheaper to implement in the daily routine, especially as warm ischemia is such a crucial parameter for the organ quality [10,11]. While five anastomoses must be sutured for the bicaval technique, only four are required for the biatrial technique. [5,6]. Consequently, warm ischemia is often prolonged by bicaval technique [5,6]. Nevertheless, as the risk for heart rhythm disorders and tricuspid regurgitation is decreased, bicaval technique is favored over biatrial today [5,6]. By implementation of the modified bicaval technique, however, we were able to reduce the warm ischemia not only compared to the regular bicaval technique by about 275, but more important also compared to the biatrial by about 23% one as indicated by the post-hoc analyses. Therefore, a modified bicaval technique offers the advantages of bicaval anastomoses regarding heart rhythm disorders and tricuspid regurgitation of the bicaval technique. Simultaneously warm ischemia is even further decreased compared to biatrial anastomoses confirming our initial hypothesis and representing the most important result of our study.

PGD is a common adverse event following HTx and is associated with a high risk for early mortality. Several risk factors for PGD exist, with graft ischemia being one of the few influenceable during the actual HTx procedure [17,18,19]. In our whole cohort, we had 66 of 238 patients (27.7%) with postoperative temporary mechanical assistance by va-ECMO which is defined as severe PGD by the International Society for Heart and Lung Transplantation (ISHLT) [17]. Although this number might be elevated compared to international registry data due to our center’s relatively liberal ECMO implantation regime, we followed the same regime in all three groups [17,18,19]. Only one in ten patients with the modified bicaval technique developed severe PGD. In contrast, approximately three of ten patients with biatrial or bicaval technique needed postoperative temporary ECMO therapy. Keeping in mind the multifactorial genesis of PGD as a potential confounder, modified bicaval technique resulted in a relative risk reduction for PGD of about 65% in our cohort.

According to the literature, the incidence of postoperative heart rhythm disorders as well as tricuspid regurgitation is increased in patients with biatrial technique compared to bicaval [5,6,7,8,9]. We observed similar effects for heart rhythm disorders in our cohort. As a consequence, we abandoned the biatrial technique. Since then, patients were either transplanted in bicaval or modified bicaval technique. Biatrial technique can cause heart rhythm disorders due to the required right atrial incision causing sinoatrial node and atrioventricular conduction disorders [20,21]. Especially the relatively high incidence of postoperative pacemaker implantation and the associated risk for pacemaker lead infections and endocarditis of the graft are a matter of concern for the biatrial technique [22].

Modifications of the bicaval technique are common and not new in the field of HTx surgery [12,13,23,24,25,26,27,28,29]. Both alterations of the anastomosis lines as well as the number of sutured anastomoses before releasing the aortic cross-clamp have been reported in the literature [12,13,23,24,25,26,27,28,29]. However, these reports are mainly limited to case reports [12,23,24,26]. In contrast, several large studies compare the biatrial and bicaval techniques [5,6,7,8,9]. However, these studies in general lack differentiation of regular or modified bicaval techniques [5,6,7,8,9]. Therefore, evidence of the comparison of biatrial, bicaval as well as modified bicaval techniques are still lacking. In addition, as several different modifications of the bicaval technique have been described, the term modified bicaval technique itself is somehow misleading, as it covers different forms of modifications [12,23,24,26,28]. In our study, we used the term to describe that the aortic cross-clamp was released after the LA, IVC and aortic anastomoses and SVC and PA were sutured during the reperfusion of the graft in beating-heart technique. Bakhashandeh and colleagues used the same modified technique in 28 patients and compared them to 30 patients with the regular bicaval technique [13]. Although the authors reported relatively long warm ischemia with about 80 min for the control and about 62 min for the modified group, their results indicate the same trends as our study regarding ischemia and graft function [13]. Other groups release the cross-clamp already after the LA and aortic anastomoses and suture IVC, SVC and PA during reperfusion [12]. Although this modification may further decrease the graft’s warm ischemia, it also may be surgically more challenging [12]. Systematic comparison of these two techniques with the regular bicaval one is also missing and should be addressed in the future to further improve the surgical procedure.

The retrospective and single center design limits the validity of the results. Although preoperative recipient donor parameters were comparable between the three groups, group sizes were unbalanced and not matched. In addition, surgeons with different experiences had operated on the reported study patients, performed anastomoses technique was chosen by surgeon’s preferences and only regular bicaval technique was performed throughout the whole study period. To overcome these biases, a multicentric randomized trial would be needed. Nevertheless, we were able to report novel insights and strengthen the available data for the modified bicaval technique improving the outcome of patients undergoing HTx. In addition, a follow-up report with a larger proportion of patients with the modified bicaval technique and a longer follow-up period that allow multivariate analyses are planned for the future.

## 5. Conclusions

The modified bicaval technique enables to decrease in the crucial warm ischemia during HTx compared to both biatrial and regular bicaval techniques. As the risk for postoperative PGD increases with the overall ischemic time of the graft, the modified bicaval technique is especially helpful for cases with long transport times. Although the results are only preliminary, we were able to report promising results for a perioperative outcome, especially decreased incidence of PGD and improved short-term survival by the modified bicaval technique. In contrast to bicaval technique, patients with biatrial anastomoses needed significantly more often permanent pacemaker implantation after the HTx. Therefore, we strongly recommend bicaval anastomoses, ideally in a modified manner.

## Figures and Tables

**Table 1:** Preoperative recipient parameters.

	Biatrial	Bicaval	Modified Bicaval	*p*-Value
Recipient Variables	(n = 37)	(n = 191)	(n = 10)	
Age, y	54 ± 10	55 ± 11	57 ± 9	0.44
Female gender, n (%)	15 (40.5)	48 (25.1)	3 (30.0)	0.15
Height, cm	173 ± 9	174 ± 8	176 ± 9	0.38
Weight, kg	78 ± 18	78 ± 15	81 ± 10	0.68
Body mass index, kg/m²	25.8 ± 5.2	25.7 ± 4.4	26.3 ± 2.7	0.73
Time on wait list, d	491 ± 947	430 ± 618	246 ± 254	0.68
High urgency wait list status, n (%)	15 (40.5)	88 (46.1)	5 (50.0)	0.80
Previous thoracic surgery, n (%)	19 (51.4)	127 (66.5)	5 (50.0)	0.14
Ventricular assist device, n (%)	16 (43.2)	102 (53.4)	3 (30.0)	0.24
COPD, n (%)	5 (13.5)	15 (7.9)	2 (20.0)	0.16
Diabetes mellitus, n (%)	7 (18.9)	45 (23.6)	2 (20.0)	0.90
Arterial hypertension, n (%)	26 (70.3)	105 (55.0)	4 (40.0)	0.13
Pulmonary hypertension, n (%)	0 (0.0)	21 (11.0)	1 (10.0)	0.06
Laboratory values				
Hemoglobin, g/dL	11.8 ± 2.2	12.0 ± 2.2	12.5 ± 1.6	0.64
Creatinine, mg/dl	1.45 ± 1.21	1.39 ± 0.92	1.25 ± 0.89	0.13
Bilirubin, mg/dL	0.93 ± 0.88	0.86 ± 0.85	0.60 ± 0.34	0.74
AST, U/L	39 ± 29	38 ± 55	32 ± 14	0.35
Lactate dehydrogenase, U/L	503 ± 737	318 ± 228	300 ± 148	0.37

Preoperative characteristics of the recipients. Comparison of biatrial (n = 37), bicaval (n = 191) and modified bicaval (n = 10) anastomoses technique. Results are presented as mean values with the standard deviation respectively percentages of the whole. COPD, chronic obstructive pulmonary disease; AST, aspartate aminotransferase.

**Table 2:** Donor data.

	Biatrial	Bicaval	Modified Bicaval	*p*-Value
Donor Variables	(n = 37)	(n = 191)	(n = 10)	
Age, y	44 ± 13	43 ± 12	42 ± 8	0.72
Female gender, n (%)	15 (40.5)	89 (46.6)	3 (30.0)	0.50
Height, cm	175 ± 9	174 ± 9	173 ± 8	0.79
Weight, kg	81 ± 16	79 ± 15	79 ± 9	0.61
Body mass index, kg/m²	26.2 ± 4.9	25.9 ± 4.7	26.3 ± 1.8	0.55
Left ventricular ejection fraction, %	61 ± 8	61 ± 9	60 ± 10	0.85
Cardiopulmonary resuscitation, n (%)	14 (37.8)	49 (25.7)	5 (50.0)	0.10
Diabetes mellitus, n (%)	6/17 (35.3)	9/71 (12.7)	0/3 (0.0)	0.09
Arterial hypertension, n (%)	9/19 (47.4)	52/103 (50.5)	1/4 (25.0)	0.74
Nicotine abuse, n (%)	23/30 (76.7)	95/160 (59.4)	6/9 (66.7)	0.19
Alcohol abuse, n (%)	9/23 (39.1)	65/158 (41.1)	4/7 (57.1)	0.72
Drug abuse, n (%)	6/24 (25.0)	14/155 (9.0)	2/7 (28.6)	0.02 ^†^
Catecholamines				
Norepinephrine, µg/kg/min	0.18 ± 0.20	0.22 ± 0.34	0.20 ± 0.33	0.64
Dobutamine, µg/kg/min	3.79 ± 1.34	3.56 ± 1.34	3.52 ± 0.96	0.92
Laboratory values				
Creatinine kinase, U/L	1345 ± 2353	1867 ± 6521	2105 ± 2727	0.36
Lactate dehydrogenase, U/L	539 ± 405	542 ± 581	558 ± 250	0.39
C-reactive protein, mg/L	170 ± 154	199 ± 282	207 ± 181	0.87
Leukocytes, 10^9^/L	19.7 ± 9.2	20.8 ± 26.7	18.1 ± 7.2	0.97

Donor characteristics before organ donation. Comparison of biatrial (n = 37), bicaval (n = 191) and modified bicaval (n = 10) anastomoses technique. Results are presented as mean values with the standard deviation respectively percentages of the whole. ^†^ Post-hoc analysis of donor drug abuse: biatrial vs. bicaval: *p* = 0.03, biatrial vs. modified bicaval: *p* > 0.99, bicaval vs. modified bicaval: *p* = 0.14.

**Table 3:** Peri-procedural data.

	Biatrial	Bicaval	Modified Bicaval	*p*-Value
Peri-Operative Variables	(n = 37)	(n = 191)	(n = 10)	
Graft ischemia				
Transport time, min	150 ± 51	151 ± 48	159 ± 37	0.47
Warm ischemia, min	62 ± 11	66 ± 15	48 ± 10	<0.001 ^†^
Total, min	212 ± 50	216 ± 49	203 ± 38	0.47
Operative procedure				
Cardiopulmonary bypass time, min	214 ± 99	267 ± 70	214 ± 44	<0.001 ^††^
Reperfusion, min	89 ± 56	136 ± 47	133 ± 48	<0.001 ^#^
Duration of catecholamine therapy				
Postoperative dobutamine, h	79 ± 43	102 ± 89	96 ± 52	0.61
Postoperative epinephrine, h	120 ± 92	153 ± 153	98 ± 44	0.83
Postoperative norepinephrine, h	235 ± 351	166 ± 188	76 ± 72	0.18
Severe primary graft dysfunction				
Extracorporeal life support, n (%)	10 (27.0)	55 (28.8)	1 (10.0)	0.53
Duration, d	11 ± 12	8 ± 7	15	0.47
Successful weaning, n (%)	9 (90.0)	39 (70.9)	1 (100.0)	0.46
Hospital stay				
Mechanical ventilation, h	155 ± 223	143 ± 185	82 ± 125	0.26
ICU/IMC stay, d	34 ± 34	22 ± 23	24 ± 25	0.04 ^##^
Postoperative hospital stay, d	61 ± 54	42 ± 29	56 ± 31	0.09
Blood transfusions				
Packed red blood cells, mL	3583 ± 4203	3606 ± 4829	2314 ± 2990	0.48
Platelets, mL	1035 ± 1783	1053 ± 2173	63 ± 166	0.10
Fresh frozen plasma, mL	5593 ± 5148	5905 ± 7334	4250 ± 4521	0.54

Perioperative data. Comparison of biatrial (n = 37), bicaval (n = 191) and modified bicaval (n = 10) anastomoses technique. Results are presented as mean values with the standard deviation respectively percentages of the whole. ICU, intensive care unit; IMC, intermediate care unit. ^†^ Post-hoc analysis of warm ischemia: biatrial vs. bicaval: *p* = 0.29, biatrial vs. modified bicaval: *p* = 0.03, bicaval vs. modified bicaval: *p* = 0.001. ^††^ Post-hoc analysis of cardiopulmonary bypass time: biatrial vs. bicaval: *p* < 0.001, biatrial vs. modified bicaval: *p* > 0.99, bicaval vs. modified bicaval: *p* = 0.06. ^#^ Post-hoc analysis of reperfusion: biatrial vs. bicaval: *p* < 0.001, biatrial vs. modified bicaval: *p* = 0.002, bicaval vs. modified bicaval: *p* > 0.99. ^##^ Post-hoc analysis of ICU/IMC stay: biatrial vs. bicaval: *p* = 0.03, biatrial vs. modified bicaval: *p* = 0.42, bicaval vs. modified bicaval: *p* > 0.99.

**Table 4:** Outcome.

	Biatrial	Bicaval	Modified Bicaval	*p*-Value
Outcome Variables	(n = 37)	(n = 191)	(n = 10)	
Postoperative morbidity				
Neurological events, %	8 (27.6)	32/189 (16.9)	1/9 (11.1)	0.72
Thoracic re-exploration, %	15 (40.5)	53/189 (28.0)	2/9 (22.2)	0.30
Acute graft rejection, %	1 (2.7)	14/189 (7.4)	1/9 (11.1)	0.37
Infections, %	10 (27.0)	43/189 (22.8)	2/9 (22.2)	0.90
Heart rhythm				
At hospital discharge				
Sinus rhythm, %	27/33 (81.8)	157/163 (96.3)	10 (100.0)	0.01 ^†^
Pacemaker, %	6/33 (18.2)	5/164 (3.0)	0 (0.0)	0.01 ^††^
At last follow-up				
Sinus rhythm, %	27/33 (81.8)	150/162 (92.6)	10 (100.0)	0.14
Pacemaker, %	6/33 (18.2)	6/162 (3.7)	0 (0.0)	0.01 ^†††^
Survival				
30-day, n (%)	35 (94.6)	169/189 (89.4)	9/9 (100.0)	0.56
1-year, n (%)	21/26 (80.8)	137/174 (78.7)	1/1 (100.0)	>0.99

Postoperative outcome and early survival. Comparison of biatrial (n = 37), bicaval (n = 191) and modified bicaval (n = 10) anastomoses technique. Results are presented as mean values with the standard deviation respectively percentages of the whole. ^†^ Post-hoc analysis of sinus rhythm at discharge: biatrial vs. bicaval: *p* = 0.01, biatrial vs. modified bicaval: *p* = 0.31, bicaval vs. modified bicaval: *p* > 0.99. ^††^ Post-hoc analysis of implanted pacemakers at discharge: biatrial vs. bicaval: *p* = 0.004, biatrial vs. modified bicaval: *p* = 0.31, bicaval vs. modified bicaval: *p* > 0.99. ^†††^ Post-hoc analysis of implanted pacemakers at last follow-up visit: biatrial vs. bicaval: *p* = 0.01, biatrial vs. modified bicaval: *p* = 0.31, bicaval vs. modified bicaval: *p* > 0.99.

## Data Availability

The data underlying this article will be shared on reasonable request to the corresponding author.
